# Incorporating Multivariate Auxiliary Information for Traffic Prediction on Highways

**DOI:** 10.3390/s23073631

**Published:** 2023-03-31

**Authors:** Bao Li, Jing Xiong, Feng Wan, Changhua Wang, Dongjing Wang

**Affiliations:** 1 Technology R&D Center, Zhejiang Institute of Mechanical & Electrical Engineering Co., Ltd., Hangzhou 310053, China; 2 School of Modern Information Technology, Zhejiang Institute of Mechanical and Electrical Engineering, Hangzhou 310053, China; 3 School of Computer Science and Technology, Hangzhou Dianzi University, Hangzhou 310018, China

**Keywords:** time series forecasting, traffic prediction, long-short term memory network, attention mechanism

## Abstract

Traffic flow prediction is one of the most important tasks of the Intelligent Transportation Systems (ITSs) for traffic management, and it is also a challenging task affected by many complex factors, such as weather and time. Many cities adopt efficient traffic prediction methods to control traffic congestion. However, most of the existing methods of traffic prediction focus on urban road scenarios, neglecting the complexity of multivariate auxiliary information in highways. Moreover, these methods have difficulty explaining the prediction results based only on the historical traffic flow sequence. To tackle these problems, we propose a novel traffic prediction model, namely Multi-variate and Multi-horizon prediction based on Long Short-Term Memory (MMLSTM). MMLSTM can effectively incorporate auxiliary information, such as weather and time, based on a strategy of multi-horizon time spans to improve the prediction performance. Specifically, we first exploit a multi-horizon bidirectional LSTM model for fusing the multivariate auxiliary information in different time spans. Then, we combine an attention mechanism and multi-layer perceptron to conduct the traffic prediction. Furthermore, we can use the information of multivariate (weather and time) to provide interpretability to manage the model. Comprehensive experiments are conducted on Hangst and Metr-la datasets, and MMLSTM achieves better performance than baselines on traffic prediction tasks.

## 1. Introduction

Time series forecasting plays an essential role in many domains, including energy, economics and finance and transportation. In recent years, traffic management departments and related researchers have begun to focus on the study of traffic flow prediction. Traffic flow prediction is a sub-field of time series forecasting, which can predict the number of vehicles at several future times based on the historical traffic data [1]. Accurate prediction for future traffic states could help departments better schedule and ensure the normal operation of the highways. In addition, traffic flow prediction could also be used for recommending more convenient paths for drivers and providing convincing information for traffic management decisions.

Many existing traffic flow prediction methods are mainly designed to focus on capturing intra-sequence temporal patterns in thousands of related time series. Traditional time-series-based methods, such as auto-regressive integrated moving average (ARIMA) and Kalman filtering, have been widely used for traffic flow prediction [2]. Based on the assumption that the time series data are stationary (meaning that the mean and variance do not change over time), ARIMA maintains the series stationarity by logarithmic transformation or difference and uses the historical data of the univariate series to predict future values. However, in most scenarios, the time series data is not only a univariate sequence. Rather, it will be associated with a variety of complex contextual information, such as spatial information, temporal information and weather information. Furthermore, some studies exploit external contextual information for better prediction. For example, Pan et al. [3] utilized the spatiotemporal information and short-term and long-term average speed on road segments for more accurate prediction, and Wu et al. [4] combined the information POIs, weather, geo-tagged tweets and collision records to predict the traffic flow. However, these methods are unable to capture the complex non-linear spatiotemporal dependencies and rely heavily on feature engineering. Overall, we argue that traditional methods lack the capability to model complex relations within one series.

Recent years have witnessed increasing interest in applying deep neural networks (DNN) in many applications (such as machine translation and image captioning), which have a great ability to capture non-linear dependency in the real world. Convolutional Neural Network (CNN) is a DNN-based model with a three-layer structure, which has the ability of data reduction and feature extraction under the action of different convolutional kernels. CNN-based models are applied to time series prediction.

Zhang et al. [5] designed a DNN-based model, DeepST, to simulate time series features. Different from DeepST, Zhang et al. [6] proposed a DNN-based approach, called ST-ResNet, to model the temporal trend properties and the spatial properties of traffic.

Compared with CNN-based methods, the Recurrent Neural Networks (RNN) consider the current input data and historical input sequence and choose to enhance or forget historical information by a memory unit or gating mechanism and other strategies. So, RNN-based models have great ability in representation learning and long-term dependencies from serialization and time series data. There are two widely used RNN-based methods, the first one is Long Short-Term Memory (LSTM), which introduces memory units based on sequence modeling to control long-term dependencies. Yu et al. [7] built a DNN-based framework based on LSTM units, which applies LSTM to forecast peak-hour traffic and identify unique characteristics of traffic data. Yao et al. [8] proposed a Deep Multi-View Spatiotemporal Network (DMVST-Net) framework to model both spatial and temporal relations. Another one is Gated Recurrent Units (GRUs), which simplify the gating mechanism of LSTM by using the relationship between the current unit information and the previous global information. Tao et al. [9] proposed a model named Selected Stacked Gate Recurrent Units (SSGRUs) to predict the traffic flow through a sparsely traveled road. It can improve efficiency and decrease computing costs. Zhao et al. [10] used the Adam optimization algorithm to optimize the weights in the GRU model to predict the travel speed of trucks on the road. Moreover, the combination of different DNN-based models can also improve the ability of prediction. For example, Liu et al. [11] combined convolution and LSTM to form a Conv-LSTM model, which can extract spatiotemporal information of the traffic flow information.

**Figure 1:**
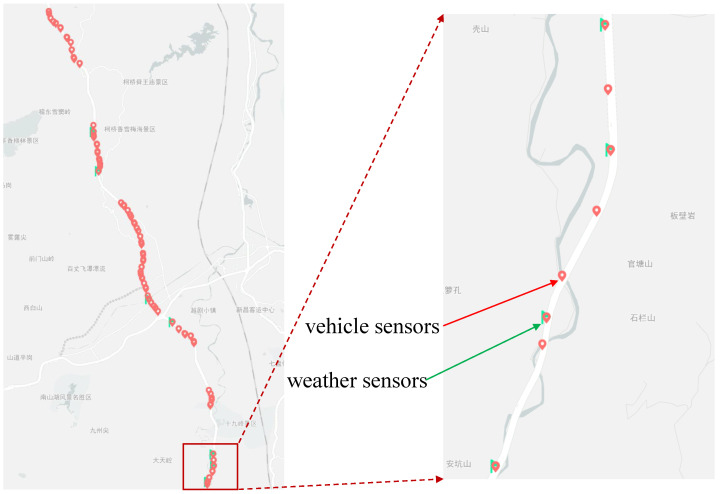
The distribution of vehicle sensors and weather sensors on Hangst highways.

Recently, researchers have proposed many traffic flow forecasting methods based on Graph Convolution Networks (GCNs) [1,12,13,14], which can fully exploit the topological information of the graph structure and the spatial correlation among sensor nodes. However, in most highway scenarios (such as the section of Hangst highway shown in Figure 1), it is difficult to construct the graph structure and employ the GCN-based models. Although these models can make effective predictions, the DNN-based models have insufficient interpretability of prediction results, and it is difficult to provide valuable analysis for the management department.

To overcome these issues, we propose a hybrid traffic prediction framework, namely Multi-variate and Multi-horizon prediction based on Long Short-Term Memory (MMLSTM) to enhance the performance of prediction and to use the multivariate information to provide interpretability to the traffic management department. This research focuses on studying (1) how to effectively capture the relations of multivariate auxiliary information in highways, and (2) how to model the effects of different time spans on historical sequences. Based on the assumption that different lengths of historical information have different impacts on the current prediction task, we design different horizons to capture the short-term dependence for a period of days and the long-term dependence for a period of weeks and use explicit ways to increase the impact of different dependencies. Specifically, we first exploit a BiLSTM model for fusing the multivariate auxiliary information (e.g., weather and time) in different time spans. Furthermore, we employ a soft attention module to integrate the different effects of different horizons. Overall, the main contributions of this work are listed as follows:

- We propose a novel BiLSTM-based model, which combines multivariate auxiliary information in highways to learn the representation of features for better prediction performance.
- We design a multi-horizon strategy and use the soft attention module to integrate the different effects of different horizons.
- We conduct comprehensive experiments on two datasets, and the results show that the proposed model MMLSTM achieves better performance than the baselines.

To more clearly illustrate the main contributions of our proposed method, Table 1 compares our approach with existing methods for two research gaps.

The rest of this work is organized as follows. We present the related works in Section 2. Section 3 gives formal definitions and preliminaries of traffic flow prediction and details of the proposed model MMLSTM. Next, we evaluate MMLSTM, compare it with some baselines and analyze the experiment results in Section 4. Last, the conclusion and future work are presented in Section 5.

## 2. Related Work

There is a long history of research on time series forecasting and traffic flow prediction tasks. Many different types of models have achieved effective results in different scenarios and have made significant contributions to industrial practice and academic research. In this section, we first introduce some existing traditional methods for time series forecasting and then review the DNN-based models and attention-based models used in traffic flow prediction.

### 2.1. Traditional Methods for Time Series Prediction

Auto-Regressive Integrated Moving Average (ARIMA) is the classical method for time series prediction, which can effectively extract the long-term dependency of time series. For example, Amini et al. [15] used the ARIMA-based method for demand forecasting of conventional electrical load (CEL) and charging demand of EV (CDE) parking lots simultaneously. Geetha et al. [16] exploited the ARIMA-based method for rainfall prediction. However, ARIMA-based methods only focus on seasonality and regularity, which ignore the spatial correlations of time series.

Matrix Factorizing (MF) is widely used in the field of recommendation systems. It uses collaborative filtering thoughts. MF-based models can also be applied to the task of time series prediction, which can capture the potential relationship among different time series. For example, Zhang et al. [17] developed an MF-based model, named Sparsity Regularized Matrix Factorization (SRMF), which leverages the sparsity of real-word traffic matrices and the spatiotemporal properties for network traffic prediction. Yu et al. [18] presented a Temporal Regularized Matrix Factorization (TRMF) framework to solve the high-dimensional time series problem for demand forecasting. Mellit et al. [19] proposed a least squares support vector machine (LS-SVM) for short-term prediction of meteorological time series.

In addition, there are methods based on the Bayesian network and the Boltzmann machine. Das et al. [20] presented a probabilistic approach based on a fuzzy Bayesian network to forecast the weather condition among different climate variables. Kuremoto et al. [21] applied multiple layers of a Restricted Boltzmann Machine (RBM) to capture the feature of input data for time series forecasting. However, these traditional methods are unable to capture complex non-linear spatiotemporal dependencies and rely heavily on feature engineering. As a result, more complex and effective prediction models based on DNN emerged.

### 2.2. DNN for Traffic Flow Prediction

Recently, DNN-based methods have been widely applied as modules of representation learning in many applications, such as image captioning and recommendation systems, which have a great ability to capture non-linear dependency in the real world. In the field of traffic flow prediction, there are also many DNN-based models, mainly including CNN-based models and RNN-based models. Many researchers employ CNN-based models to capture the correlation among different features in the task of time series prediction. For example, Zhang et al. [5] designed a DNN-based model named DeepST, which can model the features of near and distant spatial dependencies and temporal closeness, period and trend using a spatiotemporal CNN component. Different from DeepST, Zhang et al. [6] proposed a DNN-based approach, called ST-ResNet, which combines the residual neural network and convolutional neural network. Moreover, the residual neural network can solve the problem of optimization training when the number of network layers deepens. Zhang et al. [22] proposed a multitask deep-learning framework that simultaneously predicts the node flow and edge flow based on convolutional networks. Sun et al. [23] proposed a DNN-based multi-branch model called TFFNet (Traffic Flow Forecasting Network) to forecast the short-term flow throughout a city, which employs a multi-layer fully convolutional framework to perform cross-correlation calculation and extract the hierarchical spatial dependencies from local to global scales.

Additionally, the use of RNN models is prevalent in time series prediction due to their effectiveness. Yu et al. [7] built a DNN-based framework based on LSTM units. Yao et al. [8] proposed a Deep Multi-View Spatiotemporal Network (DMVST-Net) framework to model both spatial and temporal relations. Zhao et al. [10] used the Adam optimization algorithm to optimize the weights in the GRU model to predict the travel speed of trucks on the road. Tao et al. [9] proposed a model named Selected Stacked Gate Recurrent Units (SSGRUs) to predict the traffic flow through a sparsely traveled road.

Moreover, the combination of different types of models can also improve the ability of prediction. For example, Fu et al. [24] combined LSTM and GRU to predict short-term traffic flow, and Liu et al. [11] combined convolution and LSTM to form a Conv-LSTM model, which can extract spatiotemporal information of the traffic flow information. In addition, Shi et al. [25] proposed a Multiple Linear Regression and a Long Short-Term Memory (MLR-LSTM) model, which uses the incomplete traffic flow data in a past period of the target prediction section and the complete data in a past period of each adjacent section to jointly predict the traffic flow changes of the target section in a short time. Wei et al. [26] proposed a model called AutoEncoder Long Short-Term Memory (AE-LSTM), which uses AutoEncoder to capture the internal relationship of the traffic flow by extracting the characteristics of upstream and downstream traffic flow data and employs LSTM to predict the complex linear traffic flow data. Wei et al. [27] proposed a decoder convolutional LSTM model, where the convolutional operation is used to consider the correlation of the high-dimensional features, and the LSTM network is used to consider the temporal correlation of traffic flow data. Moreover, the multi-head attention mechanism is introduced to use the most relevant portion of the traffic data to improve the prediction performance.

### 2.3. Attention for Traffic Flow Prediction

The attention mechanism has also been successfully applied to fuse the feature representation. The idea of the attention mechanism is to filter a small amount of significant information from long time series data and focus on the important information [28]. Zhou et al. [29] proposed a Filter-Attention-Based Spatiotemporal Neural Network (FASTNN) to extract universal spatiotemporal dependencies from different types of historical traffic flow, and the filter-attention module can quantify the spatiotemporal aggregation of features. Moreover, FASTNN used a matrix-factorization-based resample module to automatically capture the intrinsic correlation of the same feature and reduce the redundant information between different features. Luo et al. [30] also proposed a multitask deep-learning model, which combines an attention mechanism, residual block and multi-scale convolutional network to capture complex non-linear spatiotemporal dependencies and influence factors.

## 3. Methodology

### 3.1. Traffic Flow Prediction Problem

In this part, we present the traffic flow prediction problem in detail. Traditional time series prediction scenarios have an input sequence $X=(x_{1},x_{2},\ldots ,x_{k})$ as original data, where $x_{i}$ is the temporal feature (such as price, flow). However, in the highways scenario, the input sequence is the traffic flow data. After passing the input layer, $\left(x_{(i-1)d+1},x_{(i-1)d+2},\ldots ,x_{(i-1)d+d}\right)$ can be the vector $x_{i}\in R^{d}$, where d is the aggregate window size. We choose a sliding window of length *L* to create new sequences from the original sequence X such that $X=(X_{1},X_{2},\ldots ,X_{k})$, where $X_{i}=\left(x_{i},x_{i+1},\ldots ,x_{i+L}\right),X_{i}\in R^{L\times d}$. The ground traffic flow values are given by $y=(y_{1},y_{2},\ldots ,y_{k-1})$ where $y_{i}\in R^{1}$. Our goal is to predict the next value denoted by ${\hat{y}}^{T}$. We learn a prediction model *f* by mapping the temporal sequence feature X and the corresponding ground-truth value *y* to obtain the predicted value $y^{T}$ with the following formulation,

(1) $${\hat{y}}^{T}=f\left(X,y\right).$$

### 3.2. MMLSTM

**Figure 2:**
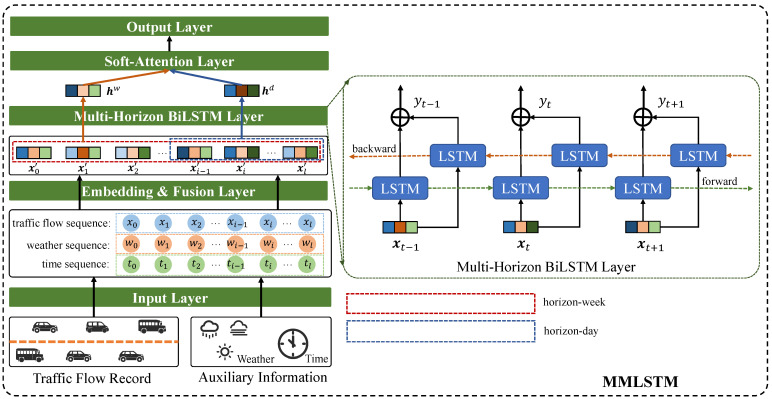
The MMLSTM model. Firstly, we put the traffic flow records and auxiliary information collected from weather and vehicle sensors into the input layer for obtaining the historical time series data. Then, we design an embedding and fusion layer for obtaining multivariate feature representation. Furthermore, considering the special scenario of highways, we proposed a multi-horizon BiLSTM layer to capture the information under different time spans (a short period of day and a long term of week), which have diverse effects on future traffic flow. Finally, we employ soft-attention to integrate two horizon information to conduct the prediction.

In this part, we present the proposed traffic flow prediction model, namely Multi-variate and Multi-horizon prediction based on Long Short-Term Memory (MMLSTM) in detail. The whole architecture of MMLSTM is shown in Figure 2.

#### 3.2.1. Embedding and Fusion Layer

In the traffic prediction scenario of highways, the highway management department uses many sensors to obtain traffic information. In addition to the traffic flow information, there are also the average speed, the traffic flow in different lanes, etc., related to the traffic flow. At the same time, according to the information timestamp recorded by the sensor, we can also obtain detailed time information such as the time, day, week and month of the current traffic flow. In addition, meteorological data such as precipitation, visibility, road slippage coefficient and other meteorological information collected by the meteorological detector of the road section will also have a certain impact on the traffic flow. Considering that the evolution of traffic flow is not only restricted by its regularity but also distributed by external weather conditions and temporal information [31], the input sequences of the model need to include external weather factors and time factors. In our highway traffic flow prediction scenario, the weather information collected by weather sensors (including road visibility, precipitation and coefficient of pavement wetness) is expressed as $X_{i}^{w}=\left(w_{i},w_{i+1},\ldots ,w_{i+L}\right)$ and $X_{i}^{w}\in R^{L\times d}$. Similarly, the temporal information is processed by the timestamp recorded by the vehicle sensors (including hour, day and week information) is expressed as $X_{i}^{t}=\left(t_{i},t_{i+1},\ldots ,t_{i+L}\right)$ and $X_{i}^{t}\in R^{L\times d}$. Therefore, the input data is based on the historical traffic flow sequence to combine with multi auxiliary information mentioned above. Compared with the traffic flow input $X_{i}$, $X_{i}^{{}^{\prime }}$ represents the input data on the time slice *i*, including traffic flow $X_{i}^{f}$, time factor $X_{i}^{t}$, and weather factor $X_{i}^{w}$, $X_{i}^{{}^{\prime }}$ can be expressed as

(2) $$X_{i}^{{}^{\prime }}=\sigma \left([X_{i}^{f}\left\|X_{i}^{t}\right\|X_{i}^{w}]W_{1}+b\right),$$

where $W_{1}\in R^{3d\times d}$ and $b\in R^{L\times d}$ represent the weight matrices and biases, $X_{i}^{{}^{\prime }}\in R^{L\times d}$, ‖ is the concatenation operation, and σ is an activation function.

#### 3.2.2. Multi-Horizon BiLSTM Layer and Attention Layer

**Figure 3:**
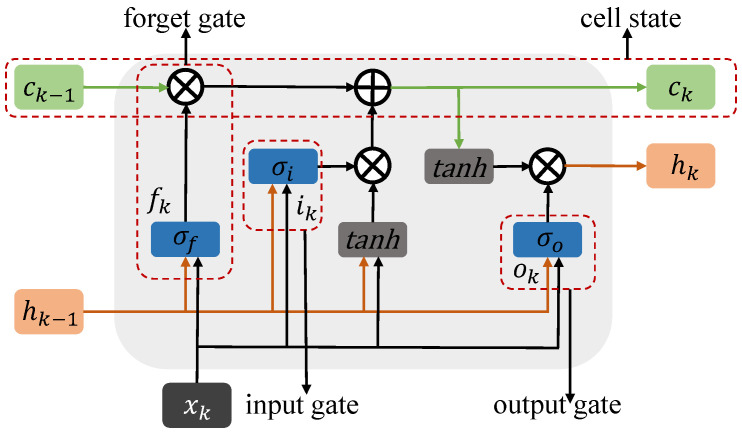
LSTM unit.

Recently, RNN-based models have great ability in representation learning and long-term dependencies captured from sequential data and are widely used for time series forecasting tasks. In the original RNN model, the information of one step is passed to the next in a built-in loop structure. However, the original RNN model suffers problems of vanishing gradients and exploding gradients. The gradients are calculated by multiplying parameter matrices, and the gradient values in distant locations become smaller, making it difficult to capture long-term dependencies in the time series. To solve these problems, the memory cell structure is introduced into the LSTM model, which contains a neuron with a self-recurrent connection and three gates. The LSTM block is shown in Figure 3. The input gate $i_{t}$ and forget gate $f_{t}$, respectively, control whether the signals of current input and previous units enter the current unit, which are expressed as follows

(3) $$i_{t}^{}=\sigma \left(W_{ii}^{}x_{t}^{}+W_{hi}^{}h_{\left(t-1\right)}^{}+b_{i}^{}\right),$$

(4) $$f_{t}^{}=\sigma \left(W_{if}^{}x_{t}^{}+W_{hf}^{}h_{\left(t-1\right)}^{}+b_{f}^{}\right),$$

where $W_{ii},W_{hi},W_{if},W_{hf}$ represent the weight matrices, $b_{i},b_{f}$ represent the biases, $x_{t}$ and $h_{\left(t-1\right)}$, respectively, represent the input feature representation of the step *t*-th unit and the output feature representation of the last step unit, and σ is the sigmoid function. For each unit of LSTM, the current state is represented by short-term memory, which is controlled by the input of the current unit. Therefore, the short-term memory ${\tilde{c}}_{t}$ is calculated as follows

(5) $${\tilde{c}}_{t}^{}=\tanh\left(W_{ic}^{}x_{t}^{}+W_{hc}^{}h_{\left(t-1\right)}^{}+b_{c}^{}\right),$$

where $W_{ic}$ and $W_{hc}$ represent the weight matrices, $b_{c}$ represents the biases, and tanh is the nonlinear activation function. The self-recurrent connection remains the long-term memory of previous units, which is updated by the input gate and forget gate, which is updated as follows

(6) $$c_{t}^{}=f_{t}^{}\times c_{\left(t-1\right)}^{}+i_{t}^{}\times {\tilde{c}}_{t}^{}.$$

The output gate determines which signals of the current unit need to be output, which is shown as follows

(7) $$o_{t}^{}=\sigma \left(W_{io}^{}x_{t}^{}+W_{ho}^{}h_{\left(t-1\right)}^{}+b_{o}^{}\right).$$

In addition, inspired by the idea of BiLSTM [32] model (shown in the right of Figure 2), we exploit a BiLSTM block to capture the periodicity and pattern of tail information on head information in sequence data. Therefore, we reverse the input sequence data and feed it into the LSTM block again to learn the reversed feature representation.

**Figure 4:**
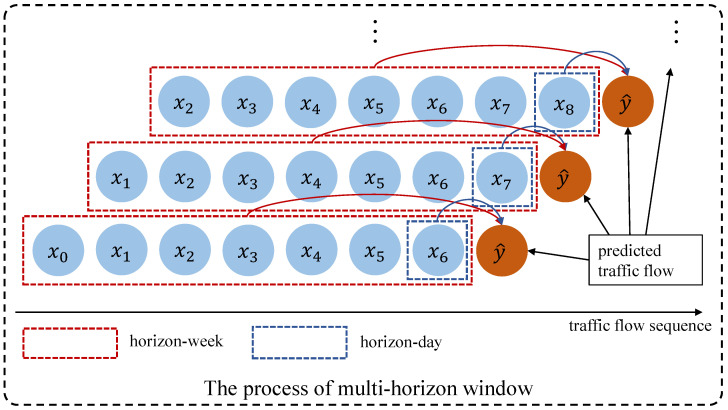
Multi-horizon window modeling.

However, the LSTM-based model captures periodic dependencies implicitly, and long-term dependencies may weaken the periodic characteristics of a certain period series, such as the periodicity of days, weeks, etc. To address this issue, we explicitly capture different periodic features based on the BiLSTM model to learn the long-term dependencies representations of sequential data from the multi-horizon. The process is shown in Figure 4. For clarity, each recording point in Figure 4 is scaled up, which is set to one day. With the increase of traffic flow records, we take the seven record points marked with red dotted lines as the horizon in a week and the record points closest to the current target to be predicted marked with blue dotted lines as the horizon in a day. We think the learned feature representation under different horizons will reflect the impact of different time spans on current predictions. In addition, we can increase or explicitly decrease the long-term dependency information.

Given a traffic flow sequence $X_{i}^{\prime }=\left(x_{1},x_{2},\ldots ,x_{T}\right)$, where *T* is the total length of a sequence, we choose a different time horizon to control the input sequence span and exploit the BiLSTM block to capture the multi-horizon dependencies. We think different span dependencies have a different impact on traffic flow prediction. Furthermore, we use a soft-attention mechanism to fuse the multi-horizon representation, which is expressed as follows

(8) $$\gamma =\frac{\exp\left(h^{\left(d\right)}\right)}{\exp\left(h^{\left(d\right)}\right)+\exp\left(h^{\left(w\right)}\right)},$$

(9) $$h^{\prime }=\gamma h^{\left(d\right)}+\left(1-\gamma \right)h^{\left(w\right)},$$

where $h^{\left(d\right)}$ and $h^{\left(w\right)}$ represent the different dependencies in the day span and the week span, γ is the weight of different horizons, which is calculated by the soft-attention mechanism, and $h^{\prime }$ is the final traffic flow feature representation.

#### 3.2.3. Traffic Flow Prediction Layer

However, we think the way other models splice each meteorological information feature based on sequence is not suitable because meteorological factors often have a greater impact on prediction at the position closest to the prediction point. Therefore, we combine representation of the final traffic flow feature z of the series with the meteorological information representation, which is expressed as follows

(10) $$z=\sigma \left(W_{2}\left[h^{\prime }\|w\right]+b_{2}\right)$$

where $W_{2}$ is a weight matrix, $b_{2}$ is bias, and w is the current weather information representation. Here, $\left[•\|•\right]$ represent the operation of concatenating two embeddings. Finally, we exploit a decoder of a full connection network to obtain the predicted traffic flow, which is derived as follows

(11) $${\hat{y}}_{i}=\sigma \left(W_{3}z+b_{3}\right),$$

where ${\hat{y}}_{i}$ represents the predicted result, $W_{3}$ is a weight matrix, and $b_{3}$ is bias. We use the squared loss function with L2-norm as the objective function, which is expressed as follows

(12) $$L^{\left(t\right)}=\sum_{t=1}^{n}\frac{1}{2}{\left[{\hat{y}}_{t}-y_{t}\right]}^{2}+\lambda {∥w∥}_{2}^{2},$$

where $y_{t}$ is the truth traffic flow, ${\hat{y}}_{t}$ is the traffic flow predicted by **MMLSTM**, and λ and w are the regularization terms for preventing model overfitting.

## 4. Experiments

### 4.1. Datasets

In this section, MMLSTM and baselines are compared on two real-world datasets, and the statistics of datasets are described in Table 2.

**Hangst.** The dataset describes the traffic flow and speed of different lanes on the Hangzhou, China highway from 1 January 2020 to 30 October 2020. It contains 200 time series and 10,560 time points. The sampling interval is 5 min.

**Figure 5:**
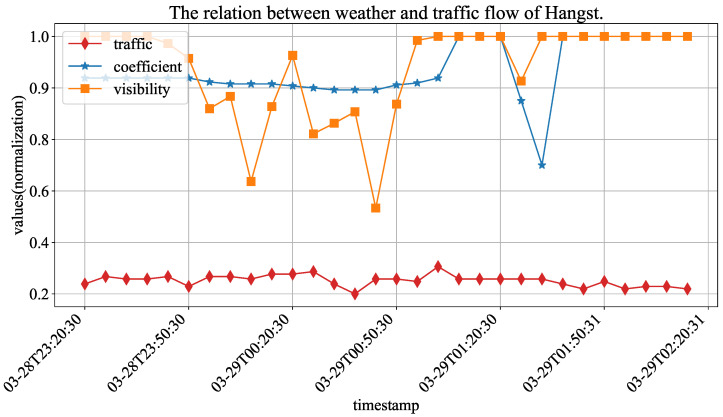
The normalized values of weather and traffic flow in Hangst.

Table 3 shows the detailed records of vehicle and weather sensors. On this highway section, two types of monitoring devices have been installed: one is the vehicle detector sensor that collects data on total traffic flow and average speed for vehicles, and the other is the meteorological sensor that collects data on visibility (road visibility), precipitation (rainfall amount) and nc_pavement_wet_coefficient (road surface slipperiness coefficient). Figure 5 shows the values of three variables (traffic flow, road visibility and road surface slipperiness coefficient). Because the scope of different variables are quite different, their values are normalized for the convenience of presentation. It can be seen that the coefficient and visibility changes and the fluctuation phenomenon of traffic flow will be enhanced. We think that the road surface slipperiness coefficient, rainfall amount and road visibility are important factors affecting driver behavior in the high-speed environment. Therefore, we choose to combine this weather information with traffic flow prediction tasks.

**Metr-la.** This dataset includes traffic information collected from sensors in the highway of Los Angeles County. It contains 207 time series ranging from 1 March 2012 to 30 June 2012 at 5 min intervals [33].

**Figure 6:**
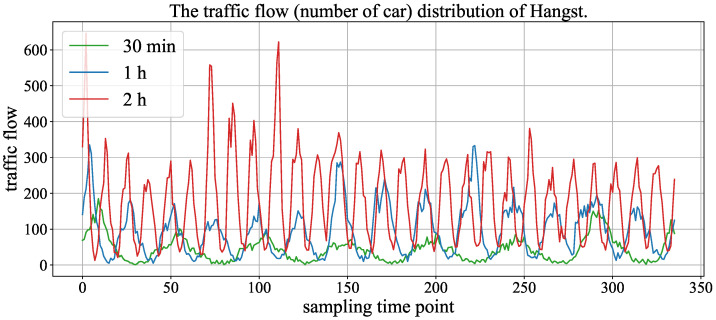
The traffic flow distribution of Hangst in different aggregation windows (time span).

**Figure 7:**
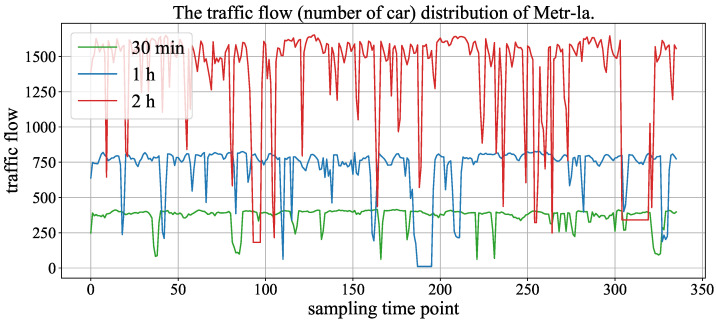
The traffic flow distribution of Metr-la in different aggregation windows (time span).

Figure 6 and Figure 7 show the traffic flow (number of cars) distribution of three aggregation window (time spans), and the signals of 30 min, 1 h and 2 h represent the size of the aggregation window (time span). In the two figures, the time axis represents traffic points collected using time windows of 30 min, 1 h and 2 h. As the number of traffic points collected varies with different time windows under the same fixed duration, we chose monthly data records with a 2 h time window to demonstrate the maximum value and also selected the same number of traffic points with 30 min and 1 h time windows from the same records. We can observe that the periodicity of the Hangst dataset is more significant than the Metr-la dataset.

For both Hangst and Metr-la datasets, after data enhancement by sliding window, we use the first 90% of sequences of each vehicle sensor as the training set and the remaining sequences as the test set. For the same sequence data, we make three predictions and finally use the average value of the three experiments as the result.

### 4.2. Evaluation Metrics

In this work, we employ two widely applied metrics, mean absolute error (MAE), and root mean square error (RMSE) for time series forecasting evaluation.

(13) $$\mathrm{RMSE}=\sqrt{\frac{1}{\xi }\sum_{j=1}^{\xi }{\left(y_{t+1,j}-{\hat{y}}_{t+1,j}\right)}^{2}},$$

(14) $$\mathrm{MAE}=\frac{1}{\xi }\sum_{j=1}^{\xi }\left|y_{t+1,j}-{\hat{y}}_{t+1,j}\right|,$$

where ξ is the total number of samples.

### 4.3. Comparison Methods

The proposed model MMLSTM is compared with six representative baselines:

- HA [34] Historical Average method forecasts the average value of the same time in the training data.
- SVR SVR is a machine learning framework based on logistic regression.
- XGBoost [35] XGBoost is a parallel regression tree model combined with boosting.
- LightGBM [36] LightGBM is a highly efficient gradient boosting decision tree.
- GRU [37] GRU introduces a gating mechanism in RNN.
- LSTM [38] Long Short-Term Memory network is an improved RNN-based model, which contains three gates to preserve the long dependencies in sequence.
- SCGRU [39] A Sparse-Connection GRU model (SCGRU) focuses on reducing the storage and computation costs using a controllable threshold on the absolute value of the pre-trained GRU weights.
- ST-Norm [40] ST-Norm contains two normalization modules that refine the local and high-frequency components of raw data and can be integrated into deep learning models such as Wavenet and Transformer.

### 4.4. Implementation and Settings

We use a grid search strategy to select the optimal learning rate and batch size. Based on our experiments, we set the learning rate to 0.001 and the batch size to 256 for both datasets. Regarding the impact of hidden size, network layer and epoch on the model, we will provide a detailed description in Section 4.5.2.

For the Hangst dataset, due to the installation of the vehicle detector sensor on this highway section in 2020, the device was unstable in the early stages of operation, leading to missing data in the monitoring data. For example, the collected traffic flow data remained at 0 for a long time. This situation occurred quite frequently in the first two months of the dataset. Therefore, we filtered the data from the first two months and used the remaining data for model training and prediction tasks. The time step of each traffic record for both datasets is set to 30 min, 1 h and 2 h.

All experiments were run on a server of Ubuntu 18.04. The server has Intel(R) Xeon(R) Silver 4108 processor, 128 GB RAM and GeForce RTX 2080Ti GPU. The Implementation is based on Python 3.7 and PyTorch 1.6.0.

### 4.5. Experiment Results

In this part, we perform comprehensive experiments to evaluate the proposed model MMLSTM and baselines in terms of accuracy by answering the following research questions:

- RQ1: Does MMLSTM outperform baseline methods in four traffic flow prediction tasks?
- RQ2: How do the value setting of parameters affect the MMLSTM’s performance?
- RQ3: Do different components of the MMLSTM improve the performance?

#### 4.5.1. Analysis of Comparative Experiment Results (RQ1)

Table 4 presents the experimental results of the highway traffic prediction for all models on different datasets. The optimal results among all methods are marked in bold, and the sub-optimal results except the proposed models, i.e., the best result among all baselines, are underlined. It can be seen that the model proposed in this paper outperforms all baselines on different datasets, which shows the effectiveness of the model.

It can be observed that our proposed model MMLSTM achieves the best performance across both datasets.

Compared with traditional models (HA and SVR), MMLSTM has a better performance because it is difficult for traditional models to process complex non-stationary time series data. For machine learning models (XGBoost and LightGBM), MMLSTM has advantages in two aspects. On the one hand, deep-learning-based models possess a greater ability for representation learning. On the other hand, traffic prediction in highway scenarios is affected by comprehensive factors, such as meteorological environment, temporal and spatial elements. MMLSTM integrates these various factors in a suitable way to capture more information for better prediction.

For RNN-based models, the RNN model learns the dependency information in time series based on a deep learning network. However, RNN lacks the ability to capture long-term dependency and has problems of gradient vanishing and gradient explosion. To solve the above problems, LSTM and GRU, respectively, introduce memory units and gate mechanisms to capture more information and have a wide range of applications in time series prediction. The experimental results in Table 4 show that LSTM and GRU perform better than RNN in most cases. Compared with LSTM and SCGRU, our model has a more accurate prediction ability. The main reason is that we have considered the complex influencing factors in highway forecasting scenarios. Although SCGRU reduces storage and computation costs, it sacrifices some prediction accuracy. Furthermore, we adaptively combine the multi-horizon time span strategy and attention mechanism to enhance the feature effect of the model.

For the ST-Norm model, the Hangst and Metr-la datasets have weak spatial interdependencies between data, which has some impact on the modeling of the ST-Norm model. However, the temporal dependence of the data is stronger, and our method is better at capturing temporal features.

In addition, it can be seen that the performance of the HA model is superior to other models in the Hangst dataset. As shown in Figure 6, we think the reason is that the Hangst dataset has a relatively obvious periodic feature.

#### 4.5.2. Analysis of Hyperparameter Experiment Results (RQ2)

First, we study the performance of MMLSTM with different embedding sizes by varying the value in the range of {8, 16, 32, 64, 128}.

**Figure 8:**
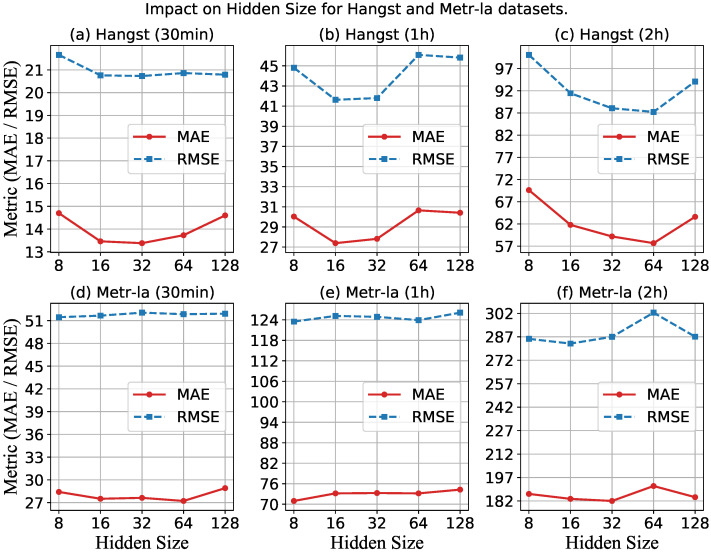
Impact of hidden size for a different dataset.

Figure 8 shows the impact of hidden size on the performance of the Hangst and Metr-la datasets. The horizontal axis represents the number of hidden sizes, and the vertical axis represents the performance metrics of MAE and RMSE. Figure 8a–c show the results of three time spans on the Hangst dataset. It can be seen that the MAE first decreases and then increases when increasing the number of hidden sizes. We think the reason is that the difficulty of calculation will be greatly increased when the hidden size is larger than a certain degree, which causes the reduction of the prediction accuracy and the problem of overfitting. Similarly, Figure 8d–f show the results of RMSE and MAE on the Metr-la dataset. Therefore, an appropriate hidden size should be set according to the actual situation.

**Figure 9:**
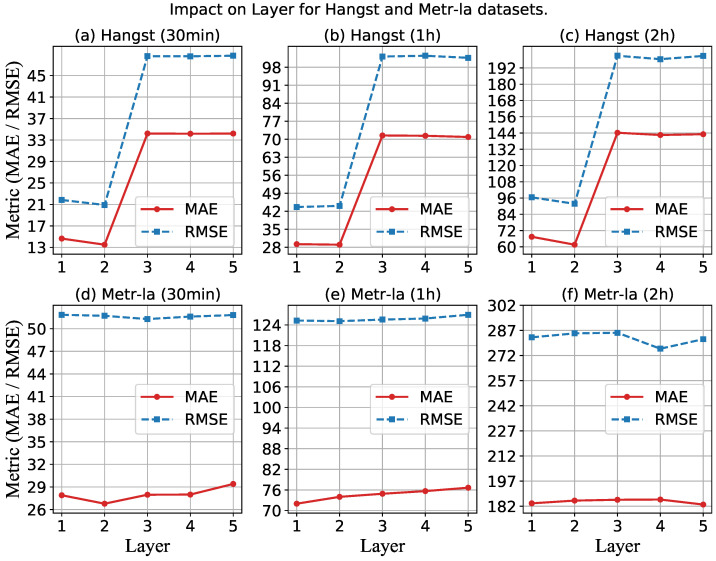
Impact on hidden layer for a different dataset.

Second, we explore the performance of MMLSTM with different layers by varying the value in the range of {1, 2, 3, 4, 5}. Figure 9 shows the impact of the layer on the performance of both datasets. For the Metr-la dataset, similar to the result of the hidden size, the effect of the network layer is not sensitive. However, the results on the Hangst dataset vary drastically with the change of the network layer. It can be seen that the metrics are the smallest when the number is two on the Hangst dataset. When the number ofthe network layer is larger than two, the performance of our model suddenly becomes worse. We think the reason is that the traffic flow of the Hangst dataset is sparser than that of the Metr-la dataset, which reflects that the traffic data value will be smaller, so it is more prone to overfitting when using deep neural networks. Moreover, from the perspective of traffic flow distribution trends, Metr-la has a more complex periodicity, so there is no phenomenon of overfitting. Therefore, when applying our model to different traffic flow prediction scenarios, more attention needs to be paid to the adjustment of the number of network layers.

**Figure 10:**
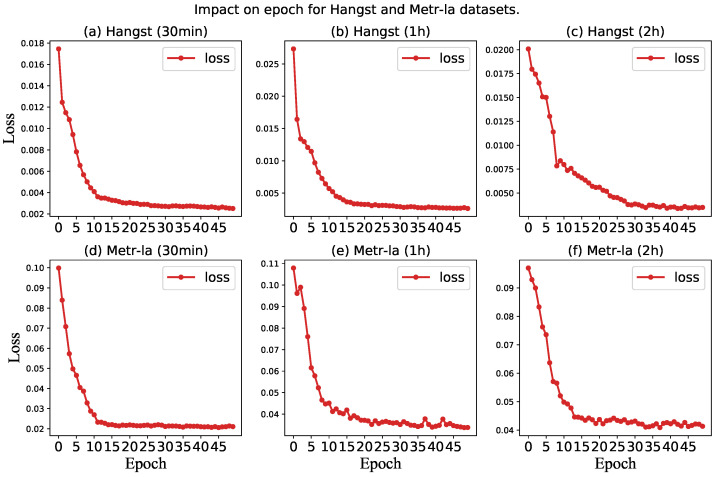
Impact on epoch for a different dataset.

Third, to study the impact of epoch on model performance, we conduct experiments to show the change curve of the loss value of the model with the increase of epoch on both datasets. The experimental results are shown in Figure 10. It can be seen from the loss curve that the MAE and RMSE of the two datasets reach the optimal value when the epoch is about 30. As a result, we have selected epoch 30 as the final parameter for our model.

#### 4.5.3. Analysis of Ablation Experiment Results (RQ3)

To study how the multivariate and multi-horizon component of MMLSTM (Ours) affects the performance, we design three variants of Ours as follows: (1) Ours-vh: This variant of the Ours model does not introduce multivariate or multi-horizon component. (2) Ours-v: This variant of the Ours model does not introduce a multivariate component. (3) Ours-h: This variant of the Ours model does not introduce a multi-horizon component.

The results are shown in Table 5 and the optimal results among all variants are marked in bold. For the Metr-la dataset, we do not have enough auxiliary information to conduct the multivariate experiments, so the ablation experiments focus on the multi-horizon component. We can observe that Ours outperforms its variants in all metrics of both datasets. The results of Ours and Ours-h of both datasets show that the learned feature representation under different horizons can reflect the impact of different time spans on current predictions. In addition, the way of increasing or decreasing the long-term dependency information explicitly can actually improve the prediction performance. Moreover, we can see a phenomenon that there is little difference between the MAE of Ours-vh (13.44, in 30 min) and Ours-v (13.78, in 30 min). We think the reason is that the influence of the multivariate is greater than the multi-horizon, so the combination of both of them can achieve better results.

#### 4.5.4. Analysis of Case Study

**Figure 11:**
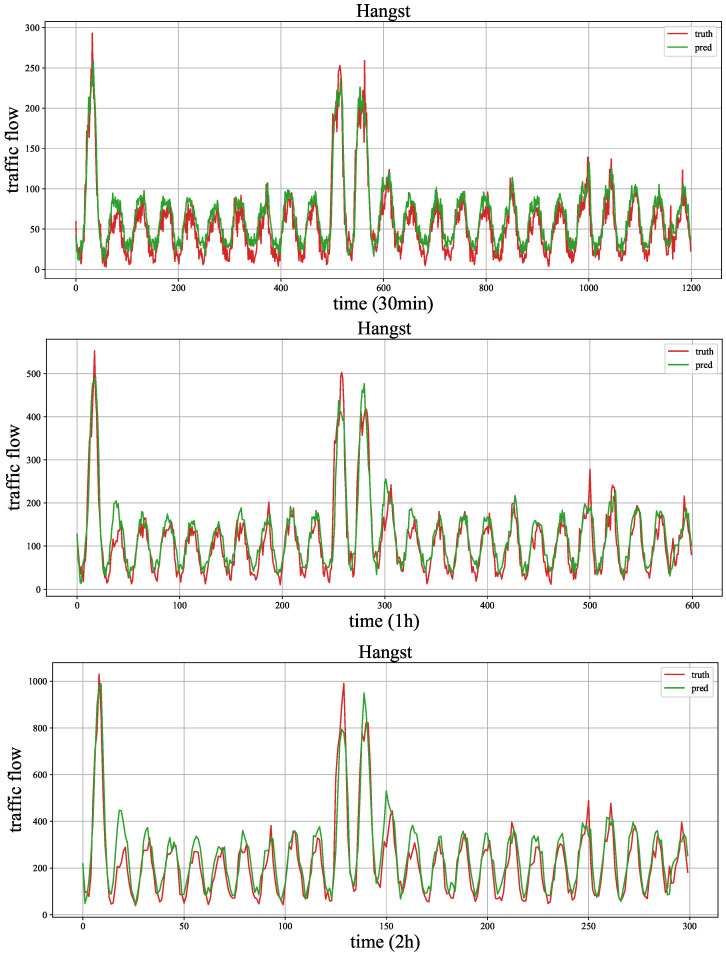
The case study of Hangst.

**Figure 12:**
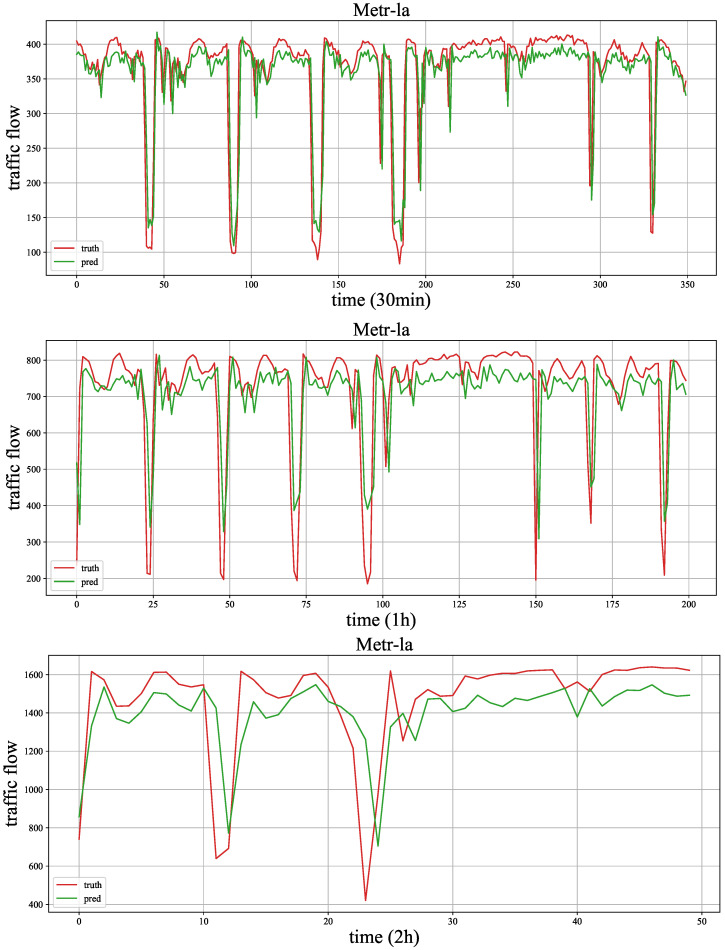
The case study of Metr-la.

As shown in Figure 11 and Figure 12, we draw the forecasting results of the proposed model MMLSTM and the truth traffic flow in three periods (30 min, 1 h and 2 h) on both datasets. We will explore the results as follows. Firstly, according to the results of the three time steps of both datasets, we can see that the fitting ability of MMLSTM can become better with the increase of time span. We think the reason is two-fold. On the one hand, short-term (30 min) traffic flow forecasts are abrupt, and it is difficult to capture the trend of drastic traffic flow changes in the future. On the other hand, it is easier to learn the periodic characteristics of time series because of the long time span (such as the traffic flow forecasting result shown in Figure 11 (2 h)). Moreover, due to the volatility of the Metr-la dataset, the predicted results are not particularly good. In practice, we should consider the data characteristics in different environments to achieve the best effect of the model.

## 5. Conclusions and Future Work

In this work, we propose a novel LSTM-based model, which combines multivariate auxiliary information with multi-horizon time spans in highways to learn the representation of features for better prediction. Specifically, based on the assumption that different lengths of historical information have different impacts on the current prediction task, we design different horizons to capture the short-term dependence with a period of days and the long-term dependence with a period of weeks and use an explicit way to increase the impact of different dependencies. Firstly, we exploit a BiLSTM model for fusing the multivariate auxiliary information (e.g., weather and time) in different time spans. Furthermore, we employ the soft attention module to integrate the different effects of different horizons. We conduct comprehensive experiments on two datasets, and the results show that the proposed model MMLSTM achieves better performance than the baselines.

However, the prediction ability of mutation points in time series forecasting is still insufficient, and the forecasting trend has a certain delay. In the future, we will alleviate the above problems based on the location and potential relationship between different sensors.

## Figures and Tables

**Table 1:** The added value of our work compared with the available literature.

Research Gaps	Available Literature	Our Work
The impact of weather factors	Mostly rely on historical traffic volume changes as the basis for traffic flow prediction and overlook the influence of meteorological factors on traffic flow prediction in high-speed scenarios	Analyze the impact of meteorological factors on traffic flow prediction in high-speed scenarios and propose a multi-dimensional auxiliary information fusion model that captures meteorological factors for traffic flow prediction
The influence of periodic patterns	Capture the periodicity in time dependence implicitly by modeling the sequence	Explicitly model the periodic changes in traffic flow, which can better capture the impact of periodic factors on traffic flow prediction

**Table 2:** Statistics of datasets.

	Hangst	Metr-la
**City**	Hangzhou	Los Angeles County
**Time span**	February–October 2020	March–June 2012
**Time Interval**	5 min	5 min
**Sensors**	200	207

**Table 3:** Sensor record examples of Hangst.

Timestamp	Device Id	Property	Value
24 March 2021 17:31:53.158+08:00	MD-TaiXing-85	total_traffic	20
24 March 2021 17:31:53.158+08:00	MD-TaiXing-85	average_speed	83
24 March 2021 17:31:53.158+08:00	WD-Mandrake-2	visibility	5000
24 March 2021 17:31:53.158+08:00	WD-Mandrake-2	precipitation	0
24 March 2021 17:31:53.158+08:00	WD-Mandrake-2	nc_pavement_wet_coefficient	65

**Table 4:** Performance comparison of highway traffic prediction.

Datasets	T	Metric	HA	SVR	XGBoost	LightGBM	RNN	LSTM	GRU	SCGRU	ST-Norm	MMLSTM
**Hangst**	30 min	RMSE	26.80	61.36	61.64	61.33	44.82	54.31	48.27	22.27	21.46	**19.98**
		MAE	22.10	43.85	42.32	42.18	29.85	45.72	37.00	17.55	17.09	**16.15**
	1 h	RMSE	48.33	122.29	121.86	122.44	100.58	87.62	96.08	46.42	40.87	**39.21**
		MAE	39.25	88.08	83.88	83.70	82.69	63.66	72.25	35.58	31.46	**30.80**
	2 h	RMSE	97.17	237.23	238.56	245.58	341.80	171.06	180.04	107.18	93.78	**87.45**
		MAE	80.00	172.59	164.17	167.61	325.04	121.58	133.42	82.64	76.06	**55.17**
**Metr-la**	30 min	RMSE	102.12	109.29	106.89	106.62	89.95	84.00	84.64	76.30	56.60	**45.20**
		MAE	73.52	70.37	66.19	66.61	44.87	60.16	59.64	55.98	26.65	**25.67**
	1 h	RMSE	191.92	248.86	240.23	232.84	196.47	194.93	198.92	177.68	140.28	**133.57**
		MAE	137.16	170.65	162.61	157.34	146.20	146.69	149.01	132.83	97.21	**89.82**
	2 h	RMSE	365.90	379.17	371.44	369.42	453.47	333.47	327.49	316.35	371.64	**304.55**
		MAE	266.37	274.11	264.44	269.61	421.48	270.01	291.38	272.00	239.58	**235.62**

**Table 5:** Ablation experiments on multivariate and multi-horizon on the Hangst dataset.

Dataset	Hangst					
***T***	**30 min**		**1 h**		**2 h**	
Metric	MAE	RMSE	MAE	RMSE	MAE	RMSE
Ours	**11.97**	**17.20**	**25.72**	**37.22**	**62.93**	**88.93**
Ours-vh	13.44	20.87	29.46	44.04	66.16	94.58
Ours-v	13.78	20.98	27.82	41.80	63.62	94.10
Ours-h	12.21	17.81	28.10	40.33	66.06	92.19
**Dataset**	**Metr-la**					
***T***	**30 min**		**1 h**		**2 h**	
Metric	MAE	RMSE	MAE	RMSE	MAE	RMSE
Ours	**27.51**	**51.66**	**74.27**	**126.10**	**182.01**	**286.97**
Ours-h	28.39	51.71	75.86	125.92	193.37	301.02

## Data Availability

The data used to support the findings of this study are available from the corresponding author upon request.
